# Practicing Partnered Research

**DOI:** 10.1007/s11606-014-3046-z

**Published:** 2014-10-30

**Authors:** Joe V. Selby, Jean R. Slutsky

**Affiliations:** Patient-Centered Outcomes Research Institute (PCORI), 1828 L Street, NW 9th Floor, Washington, DC 20036 USA

Health care research would be more relevant and have more impact if key stakeholders were invited to participate in the research process.^1^^–^ 3 Similarly, the concept and practice of the learning health care system calls for close participation in the research and development process by researchers with organizational leadership and on-the-ground clinicians, as well as patients and caregivers, to enhance the adoption of innovative discoveries .^4^^,^ 5 Research that is embedded and integrated within a working health care system can provide both generalizable knowledge and immediate value for the system and its participants by stimulating innovation and improving health outcomes, quality of care, and participant satisfaction.

The Patient-Centered Outcomes Research Institute (PCORI) has promoted the concept and practice of ongoing stakeholder “engagement” in all of its funded research – from selecting and formulating the research questions, to planning and conducting the study, analyzing results, and then disseminating and implementing study findings if warranted.^6^^,^ 7 Engagement with patients and embedding of research in delivery systems is required of nearly all studies. Involvement of non-patient stakeholders depends on the research question and target audience, but is also required in all PCORI-funded studies.

This issue of the *Journal of General Internal Medicine* focuses on “partnered research,” and we see no substantive differences between the notions of engaged research and partnered research. Currently, literature searches for either term almost exclusively yield papers concerned with patient or community involvement. This likely reflects the earlier achievements of community-based participatory research, underpinning the concept of engagement in patient-centered outcomes research.^8^^,^ 9

The papers in this *JGIM* supplement, however, focus primarily on the partnering of researchers with clinicians and health system leaders. We appreciate the opportunity to emphasize this critical aspect of engagement. Patient-centered care is a partnership involving the patient, their clinician, the delivery system, and sometimes payers or purchasers. The research activity supporting patient-centered care should be no less partnered. Greater engagement of both funding agencies and funded researchers with clinicians (physicians, nurses, pharmacists, and others) as well as health care delivery system and health plan leaders is sorely needed. These decision-makers, just as much as patients, bring important insights to the formulation of research questions, the conduct of research, the interpretation of results, and the implementation of robust findings. Together with patients, clinicians and system leaders form a community to which many of the principles of community-based participatory research are highly applicable.^10^ The phrase “nothing about us without us,” often attributed to the disability advocacy movement, is pertinent to all participants in the health care community. The Veterans Health Administration, with its highly integrated nature, has long been a pioneer and thought leader in this area.^11^^,^ 12

There are challenges to forging effective, lasting partnerships among researchers, clinicians, and health system leadership, including differences in culture, training, incentives, and priorities, as well as the language used to describe underlying perspectives. Researchers are trained to value a deliberative approach, with opportunities to carefully explore data and to fully understand research findings, methodological innovations, and data curiosities. They are driven by the need to publish and to obtain and preserve research funding. They are less comfortable focusing on observed point estimates (i.e., differences) than on the remaining uncertainty around those estimates as expressed through confidence intervals, which can frustrate decision-makers.^13^ Clinicians, by contrast, are driven to find answers to the practical questions they face with their patients. Most system leaders are focused primarily on factors that impact financial viability, including growing demands for performance measurement, the work necessary to improve performance, and means of gaining efficiency in health care delivery. Without partnership, the questions important to research funders and individual researchers may not be of interest to clinicians or health system leaders.

Time may be the most precious commodity to both clinicians and system leaders. Clinicians experience time pressures due to ever-more complex patients, transitions to electronic health records, and the growing demands of performance measurement and performance improvement. Leaders demand understandable results that are actionable, but they may not fully appreciate the importance of careful data collection, analysis, and communication. These discrepancies are often cited as barriers to more effective partnership and better use of available health care data. Importantly, the model of the self-contained research project, in which data are sequestered and analysis postponed until the end of the study period, must be augmented by rapid, iterative approaches to data analysis that enable all parties to learn and adapt as quickly as possible.

The current demands on physicians and system leaders for delivery of care suggest that the changes needed for partnering must start with researchers and funders of research. The notion of the intrepid researcher pursuing an intellectual passion and building a successful career based on publications and grantsmanship in a narrow area of interest must be reexamined and adapted to a paradigm that emphasizes both relevance and responsiveness. The successful clinical or health services researcher must bring methodological skills in effectiveness or implementation research, along with good interpersonal skills to serve on multidisciplinary teams that may include organizational engineers and informaticians as well as key stakeholders. Research training should include extensive exposure to the health care delivery system such that the researcher is comfortable communicating to patients, clinicians, and members of the “C-suite.” At this point, training opportunities that embed researchers in health care delivery systems are rare. Several joint MBA/MPH degree programs have been launched in recent years, and clinicians make up a sizeable fraction of enrollees, but it is unclear how many graduates choose positions as researchers. We are unaware of post-doctoral clinical research training experiences that place trainees within delivery systems and focus specifically on system change or management.

Whether such researchers, when trained, would be best located within academic institutions or within delivery systems is an interesting question. To date, few systems have invested substantially in internal research and development activities. Academic institutions would have to change promotion practices to recognize and support the need for researchers to spend time working with system-based partners, cutting into time available for preparing publications and teaching activities. Tenure-track reward systems would have to change to incorporate the utility of publications in addition to their numbers, and to value contributions resulting in care delivery and outcomes improvement as much as publication output.

Research funding agencies will have to better understand the true information needs of clinicians and delivery systems, and will need to design funding opportunities that directly address these questions. Funders of research training programs must think creatively about how to design competitive training opportunities that place young and mid-career researchers into health care systems where they are perceived as valuable to those systems. Inclusion of more clinicians and system leaders in study sections and representation on boards or councils of funders will be needed. Clinicians and system leaders will also have to adjust priorities to partner effectively with researchers and to take advantage of the data now accumulating in their clinical and administrative data systems. As systems become more familiar with their data through research, partnerships should look to identify and use findings as early as possible in the research project. From the clinician or system leader perspective, unexpected findings may often be the most valuable part of a research project, leading to practice or policy changes locally as well as to generalizable knowledge.

There is also a great need for systems to address present concerns about joining data from two or more institutions – even of competitors – for purposes of addressing specific research questions, especially when the research questions have been judged as important. The use of distributed research network approaches that leave data in place or transfer the minimum data needed to support analysis can help to protect data security and privacy as well as each organization’s proprietary interests.^14^ Multi-system partnerships enable larger studies, novel studies that take advantage of variations in practice and address a broader range of critical questions, and which can yield more definitive, reliable, and persuasive results.

To further enhance the value of partnered research, both researchers and health systems will need to embrace more transparent and open approaches to conducting and reporting research.^15^^,^ 16 Prompt posting of study protocols on publicly accessible websites such as ClinicalTrials.gov, publishing results whether positive or negative, and sharing of data are as important in comparative effectiveness and health system research as in the basic sciences, if findings are to be trusted by those who must disseminate and implement them. The intersection of patient-centered research, the arrival of accessible, clinically rich patient-level data, and the vision of rapid-learning health care systems promises health care we can trust, take pride in, and pay for. Neither researchers nor health systems can achieve this goal without the other, but major cultural changes will be necessary in both.
